# Reusing a prepaid health plan’s fecal immunochemical tests for microbiome associations with colorectal adenoma

**DOI:** 10.1038/s41598-022-18870-w

**Published:** 2022-08-31

**Authors:** James J. Goedert, Zhenyi Wu, Cyndee H. Yonehara, Timothy B. Frankland, Rashmi Sinha, Gieira S. Jones, Yunhu Wan, Jacques Ravel, Ni Zhao, Stacey A. Honda

**Affiliations:** 1 Division of Cancer Epidemiology and Genetics, National Cancer Institute, National Institutes of Health, Bethesda, MD USA; 2 Department of Biostatistics, Bloomberg School of Public Health, The Johns Hopkins University, Baltimore, MD USA; 3 Center for Integrated Health Care Research, Kaiser Permanente Hawaii, Honolulu, HI USA; 4 Institute for Genome Sciences, University of Maryland School of Medicine, Baltimore, MD USA; 5 Hawaii Permanente Medical Group, Honolulu, HI USA; 6 Washington, USA

**Keywords:** Biomarkers, Epidemiology

## Abstract

An altered colonic microbiota probably increases colorectal adenoma (CRA) and cancer (CRC) risk, but large, unbiased fecal collections are needed to examine the relationship of gut microbiota diversity and composition to colorectal carcinogenesis. This study assessed whether fecal immunochemical tests (FITs) from CRA/CRC screening may fulfill this requirement. Using FIT, self-collected by members of Kaiser Permanente Hawaii (KPH), as well as interspersed quality control (QC) specimens, DNA was extracted and amplified to generate 16S rRNA microbiome profiles rarified at 10,000 reads. CRA/CRC were diagnosed by colonoscopy and histopathology. Covariates were from electronic KPH records. Of 921 participants’ FIT devices, 538 (58%) yielded at least 10,000 rRNA reads and 1016 species-level variants mapped to 46 genera. Of the 538 evaluable participants, 63 (11.7%) were FIT-negative per protocol, and they were considered negative for CRA/CRC. Of the 475 FIT + participants, colonoscopy and pathologic review revealed that 8 (1.7%) had CRC, 71 (14.9%) had high-risk CRA, 107 (22.5%) had low-risk CRA, and 289 (60.8%) did not have CRA/CRC. Men were 2.27-fold [95% confidence interval (CI) 1.32–3.91] more likely than women to be FIT+ . Men also had 1.96-fold (CI 1.24–3.07) higher odds of low-risk CRA, with similar trends for high-risk CRA and CRC. CRA/CRC were not associated with overweight, obesity, diabetes, or antibiotic prescriptions in this study. QC analysis across 24 batches of FIT devices revealed QC outliers in four batches. With or without exclusion of the four QC-outlier batches, as well as lenient (1000-read) rarefaction, CRA/CRC had no consistent, statistically significant associations with fecal microbiome alpha diversity, beta diversity or genera relative abundance. CRA/CRC had expected associations with male sex but not with microbiome metrics. Fecal microbiome profiling using DNA extracted from at-home collected, re-used FIT devices is feasible, albeit with substantial challenges. Using FITs for prospective microbiome studies of CRA/CRC risk should consider the impact of the current findings on statistical power and requisite sample sizes.

## Introduction

In the United States (US), colorectal cancer (CRC) is the third leading cause of cancer death for both men and women. Screening for CRC can reduce this mortality by excision of early-stage CRC or its precursor, colorectal adenoma (CRA). In May 2021, the US Preventative Services Task Force (USPSTF) published recommendations for CRC screening of asymptomatic adults age 45 and older who are at average risk (i.e., excluding those with previous CRC, adenomatous polyps, inflammatory bowel disease, familial adenomatous polyposis, Lynch syndrome, or other inherited disposition to CRC)^1^. When employed as recommended, the USPSTF deemed screening with stool-based tests or direct visualization tests to have substantial net benefit from ages 50–75, moderate net benefit for ages 45–49, and small net benefit for ages 76–85. The recommended stool-based tests, which require no bowel preparation, include the high-sensitivity guaiac fecal occult blood test and fecal immunochemical test (FIT) annually, as well as the stool DNA-FIT (every 1–3 years). Positive results on stool tests require follow-up with colonoscopy. The direct visualization tests, which require bowel preparation prior to screening, include computed tomography (CT) colonography (every 5 years); flexible sigmoidoscopy (every 5 years; every 10 years conditional on annual FIT); and colonoscopy (every 10 years). Abnormalities detected by flexible sigmoidoscopy or CT colonography require follow-up with colonoscopy. No screening test has perfect sensitivity and specificity. For a single FIT (i.e., not annual), USPSTF summarized sensitivity for CRC detection as 0.74 (specificity for no CRC, 0.94), and sensitivity for high-risk CRA as 0.23 (specificity for no high-risk CRA 0.96)^1^. Based on such estimates, many large health systems use annual FIT, plus follow-up colonoscopy, as primary CRC/CRA screening to maximize net benefit for their hundreds of thousands of members^2^.

Although current CRC/CRA screening is clearly beneficial, it also is imperfect and cumbersome. One advance, detection of CRC-associated human DNA alterations in stool^3,4^, has lengthened the screening interval to as long as 3 years. Another approach would be to examine microbes in the stool. Over the past decade, a substantial body of research has found that the distal gut’s microbial population (the microbiota), as represented by its aggregate genome (the microbiome), may contribute to the development of CRC and CRA^5^. Postulated mechanisms include microbial metabolism (generation of pro- versus anti-carcinogens) and particularly interactions with host immunity that result in inflammation and increased mitosis of the colorectal mucosa^6,7^. The composition and diversity of the distal gut microbiota and its association with CRC^8–11^, CRA^7,12–16^, diabetes^17^, obesity^18^, and other conditions can be estimated by DNA amplification and sequencing analysis of the fecal microbiome.

A major obstacle to determining whether characterization of the fecal microbiota could be employed as a CRC/CRA screening modality has been the lack of efficient, inexpensive, and unbiased methods for the collection and storage of feces from large groups of people. Re-use of FIT devices might be an effective solution. In laboratory-based comparisons, FIT-based microbiome metrics have been nearly equivalent to metrics based on immediately frozen or chemically preserved feces^19–21^.

This project assessed whether re-use of FIT devices, immediately frozen after detection of hemoglobin, would reveal characteristics of the fecal microbiome associated with CRC, high-risk CRA, or low-risk CRA in the Kaiser Permanente Hawaii (KPH) health system.

## Results

### Study population

**Figure 1:**
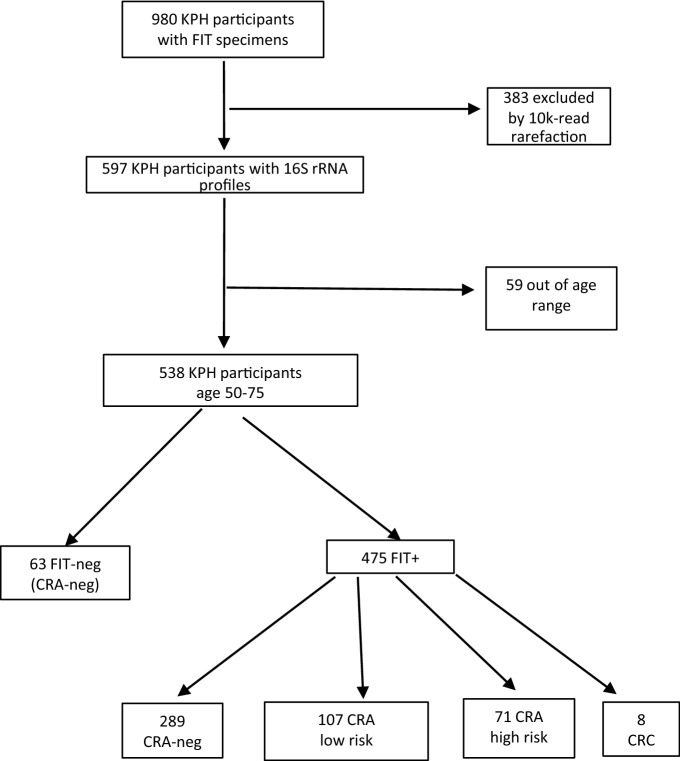
Flow chart of specimens, colorectal adenoma (CRA), colorectal carcinoma (CRC), and fecal immunochemistry test (FIT) status of Kaiser Permanente Hawaii participants. High-risk CRA had diameter ≥ 1 cm or villous histology.

As shown in Fig. 1, FIT devices were provided by 980 KPH participants, kept frozen, and subsequently subjected to DNA extraction, 16S rRNA amplification, and sequencing. Of these, 383 had fewer than 10,000 16S rRNA sequences (our rarefaction criterion), and 59 other participants were outside the per-protocol age range, leaving 538 participants for association analyses. As per protocol, 63 (11.7%) of the 538 participants were FIT-negative, and they were considered negative for CRA/CRC. Of the 475 FIT + participants, colonoscopy and pathologic review revealed that 8 (1.7%) had CRC, 71 (14.9%) had high-risk CRA, 107 (22.5%) had low-risk CRA, and 289 (60.8%) did not have CRA/CRC.

The analyzed population comprised 292 men and 246 women, with mean age 62 years (range 50–75 years). They included 280 self-described as multi-racial, 175 Whites, 55 Asians (mostly Japanese/Chinese), and 28 Other race/ethnicity. Measured weight and height revealed that 203 (37.7%) were obese (BMI > 30 kg/M^2^) and 161 (29.9%) were overweight (BMI 25–29.99 kg/M^2^). Type 2 diabetes mellitus had been diagnosed in 143 (26.6%). Charlson index was zero (no serious comorbidity) in 506 (94%). During the 365 days before collection of the FIT specimen, 87 participants (16.2%) had been prescribed an antibiotic for > 10 days; 115 (21.4%) for ≤ 10 days; and 336 (62.5%) had no antibiotic prescription.

### Demographic and clinical associations

Table 1 presents associations of FIT+, low- and high-risk CRA, and CRC with sex, overweight, obesity, diabetes and days with an antibiotic prescription in the previous year. Men were 2.27-fold (95% CI 1.28–4.10, *P* < 0.05) more likely than women to be FIT+. Men also had 1.95-fold (CI 1.22–3.16, *P* < 0.05) higher odds of low-risk CRA, with similar trends for high-risk CRA (OR 1.36, CI 0.81–2.27, *P* = 0.31) and CRC (OR 2.56, CI 0.45–25, *P* = 0.30), or CRA/CRC combined (OR 1.84, CI 1.26–2.69, *P* < 0.01). CRA/CRC was not associated with overweight, obesity, diabetes, or antibiotic prescriptions among the participants. No participant had Crohn’s disease or ulcerative colitis.

**Table 1:** Associations of phenotypes (*) FIT + , low- and high-risk CRA, and CRC with independent variables—sex, diabetes, body mass index (BMI), and antibiotics (ABX) in previous year.

Variables:	Female	Male	BMI < 25	BMI 25–29.9	BMI 30 +	No diabetes	Diabetes	ABX^b^ = 0	ABX^b^ 1–10d	ABX^b^ > 10d
Total	246	292	174	161	203	395	143	336	115	87
FIT− (n)	40	23	16	20	21	47	16	43	13	7
FIT+* (n)	206	269	109	141	182	348	127	293	102	80
Odds ratio	Ref	**2.27**	Ref	1.03	1.27	Ref	1.07	Ref	1.15	1.68
95% CI		**1.28–4.10**		0.48–2.21	0.59–2.68		0.57–2.10		0.58–2.43	0.71–4.58
*P*-value		** < 0.05**		1.00	0.59		0.88		0.75	0.26
FIT-/CRA- ^a^(n)	179	173	90	98	130	256	96	225	70	57
CRA low risk* (n)	37	70	23	35	48	75	32	57	27	23
Odds ratio	Ref	**1.95**	Ref	1.39	1.44	Ref	1.14	Ref	1.52	1.59
95% CI		**1.22–3.16**		0.76–2.54	0.82–2.54		0.68–1.87		0.86–2.66	0.86–2.89
*P*-value		** < 0.05**		**0.27**	0.20		0.62		**0.12**	**0.13**
FIT-/CRA-/CRA low risk (n)	216	243	113	133	178	338	129	289	97	81
CRA high risk*(n)	28	43	18	26	23	57	14	47	18	6
Odds ratio	Ref	1.36	Ref	1.23	0.81	Ref	0.64	Ref	1.14	0.46
95% CI		0.81–2.27		0.64–2.35	0.41–1.56		0.43–1.22		0.59–2.12	0.15–1.12
*P*-value		0.23		0.53	0.54		0.19		0.65	0.10
FIT-/CRA- (n)	179	173	90	98	130	263	97	232	70	58
All CRA* (n)	67	119	45	63	73	132	46	104	45	29
Odds ratio	Ref	**1.83**	Ref	1.24	1.12	Ref	0.94	Ref	1.43	1.12
95% CI		**1.27–2.64**		0.77–2.01	0.71–1.78		0.61–1.45		0.90–2.27	0.65–1.89
*P*-value		** < 0.01**		0.37	0.62		0.84		0.11	0.70
FIT-/CRA-/CRC- (n)	244	286	131	159	201	388	142	329	115	86
CRC* (n)	2	6	4	2	2	7	1	7	0	1
Odds ratio	Ref	2.56	Ref	0.41	0.42	Ref	0.39	Ref	0	0.55
95% CI		0.45–25		0.07–2.28	0.04–3.00		0–3.09		0–2.02	0.01–4.35
*P*-value	–	0.30		0.29	0.42		0.69		0.20	1.00
FIT-/CRA-(n)	179	173	90	98	130	256	96	225	70	57
CRA/CRC* (n)	67	119	45	63	73	139	47	111	45	30
Odds ratio	Ref	**1.84**	Ref	1.28	1.12	Ref	0.90	Ref	1.30	1.07
95% CI		**1.26–2.69**		0.79–2.07	0.71–1.78		0.59–1.38		0.82–2.06	0.62–1.80
*P*-value		** < 0.01**		0.30	0.62		0.682		0.26	0.80

Significant associations are highlighted in bold.

^a^Control group for CRA and CRC cases: FIT + without CRA or CRC, plus FIT-negatives.

^b^ABX: antibiotics use.

### Associations of microbiome composition with FIT and CRA/CRC status

After rarefaction at 10 K reads, 538 devices (61%) yielded 1016 species-level amplicon sequence variants (ASVs) mapped to 46 genera. Table 2 shows the association between microbiome alpha diversities (number of species, Chao1, Shannon index, PD-whole tree) and FIT status. Overall, the number of species, chao1 index and Shannon index were significantly lower in FIT + compared to FIT− (*P* < 0.05). The FIT + group also had lower PD-whole tree, but this was not statistically significant (*P* = 0.12). When adjusted for age, gender and race/ethnicity, there was no significant association between any of the alpha diversities and FIT status (linear regression, all *P* > 0.05). We also compared the high risk CRA/CRC group with the rest of the participants, and there was no significant difference between groups for any of the alpha diversities. There was a trend of higher alpha diversities (number of species, Chao1 and Shannon index) for CRA + compared to CRA- with a borderline significance (0.05 < *P* < 0.1). For PD whole tree, the trend was the same, but not significant. The same trend existed for high risk CRA/CRC, however, the *p*-values were not significant, possibly due to the small sample size.

**Table 2:** Fecal microbiome richness and alpha diversities by FIT and CRA/CRC status, and Chi-square *p*-values.

	FIT−	FIT+				*P*-values^a^		
		CRA-neg	CRA low risk	CRA high risk	CRC	FIT− vs FIT+	CRA− vs CRA+	High risk CRA/CRC vs Other
#of Samples	63	289	107	71	8			
**Alpha diversity: mean (SD)**								
# of species	111.75 (42.19)	95.33 (41.63)	105.00 (49.63)	99.53 (41.22)	148.94 (113.74)	** < 0.05**	0.07	0.40
Chao1	113.29 (43.76)	96.45 (43.36)	106.58 (51.55)	100.52 (42.90)	153.12 (120.46)	** < 0.05**	0.07	0.42
Shannon	5.31 (0.59)	5.00 (0.74)	5.13 (0.72)	5.13 (0.61)	5.27 (1.36)	** < 0.05**	**0.05**	0.40
PD	8.69 (2.45)	7.97 (2.54)	8.35 (2.72)	8.29 (2.91)	10.29 (3.14)	0.12	0.16	0.30

^a^Linear regression. Bold indicates statistical significance (*P* < 0.05).

**Figure 2:**
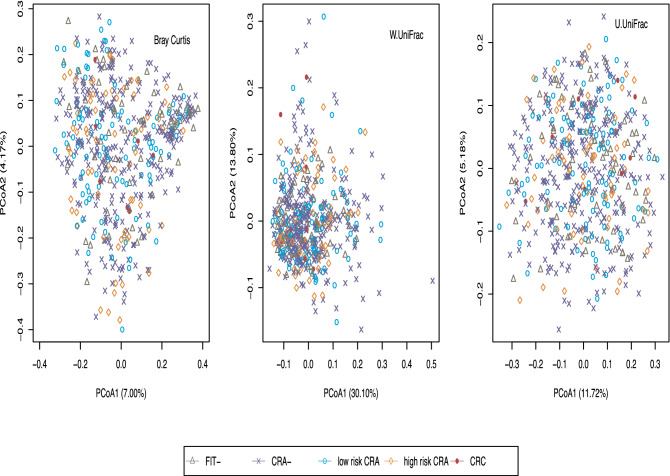
Principal coordinate plots of microbiome communities across FIT, CRA, and CRC groups.

Beta diversity was calculated using the rarefied microbiome data. Figure 2 shows the principal coordinate plots for three beta diversity measurements: Bray Curtis, weighted UniFrac (W.UniFrac) and unweighted UniFrac (U.UniFrac). Microbiome composition did not separate by disease category. We used MiRKAT to statistically assess the association between microbiome beta diversity and clinical factors. CRA + status was marginally associated with Bray–Curtis dissimilarity (*P* = 0.038) but not with U.UniFrac (*P* = 0.904) or W.UniFrac (*P* = 0.594), Omnibus (*P* = 0.101). Likewise, FIT + status was marginally associated with Bray–Curtis (*P* = 0.071) and U.UniFrac (*P* = 0.067) but not with W.UniFrac (*P* = 0.409), Omnibus (*P* = 0.167). When adjusted for BMI, age, gender and race, there were no significant associations between microbiome and any status of interest.

### Individual taxa analyses

**Figure 3:**
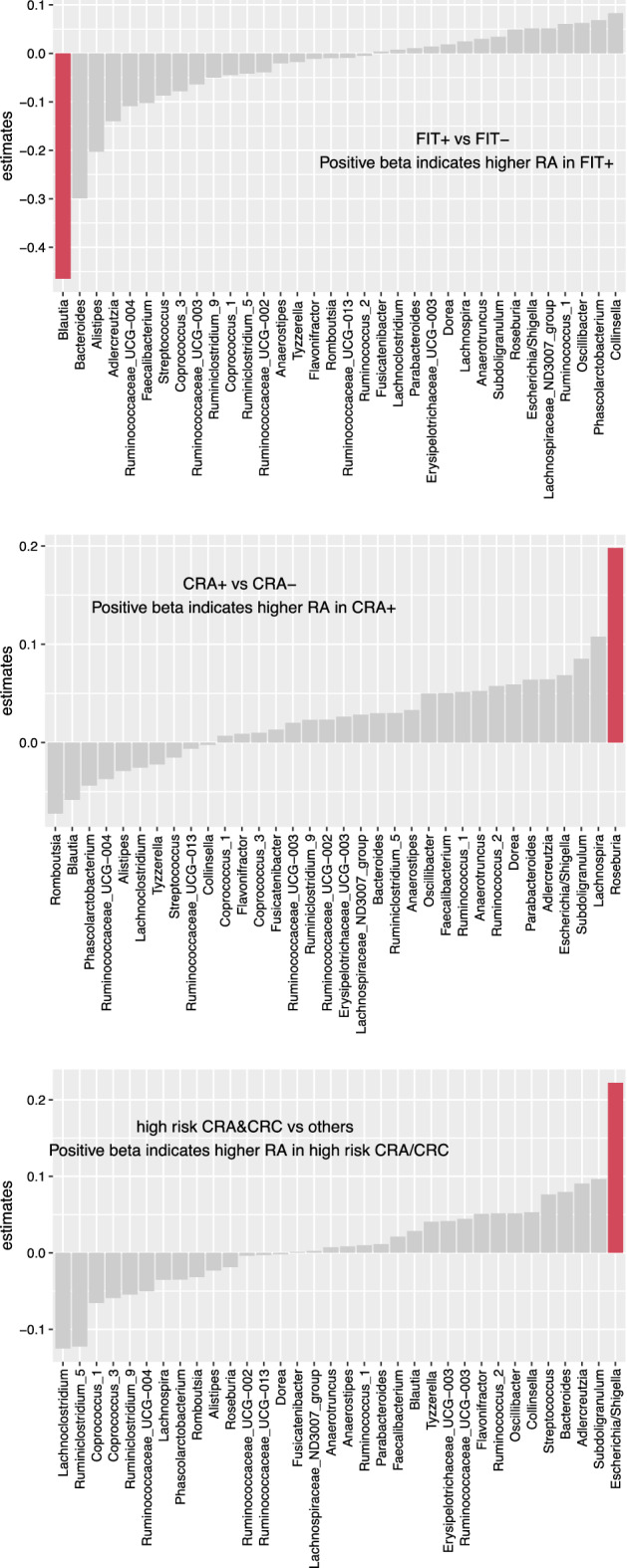
Bar plots of estimated coefficients for individual genera relative abundance by diagnosis status. Red bars indicate a nominal significant result at *p*-value < 0.05 level in unadjusted linear regression.

We evaluated the relative abundance of individual taxa at the genus level. Via unadjusted linear regression, three genera were significant: *Blautia* was significantly reduced in FIT + participants, and *Roseburia* was significantly increased in CRA + participants. Also, the relative abundance of *Escherichia/Shigella* was increased with high-risk CRA/CRC. However, none of these associations was significant at FDR 0.05 level. Figure 3 shows the beta coefficients of the associations ordered by effect size. Repeating these linear regressions after adjusting for age, gender and race, no statistically significant associations were observed at FDR 0.05 level.

### Performance of FIT devices for microbiome assessment

Among the 980 KPH participants who provided FIT devices, 102 were out of range on age. Of the remaining 878 FIT-based fecal specimens, 538 (61.2%) yielded more than 10,000 microbiome sequences, a relatively stringent rarefaction criterion that we employed for our primary analyses. With less stringent rarefaction, 650 (74.0%) specimens yielded more than 5000 reads and 811 (92.4%) specimens had more than 1000 reads. As shown in Table S1, there was no association between library size and age, gender, race, FIT status or CRA/CRC diagnosis.

### Batch effects of patient specimens and quality control (QC) samples

All FIT-based fecal specimens, together with 72 QC samples, were assigned and processed through 24 different batches for microbiome sequencing. We assessed the possibility of batch effects by first investigating systematic differences in the microbiome data. As shown in Figure S1 and Figure S2, there was no difference across the batches on sequencing depth (one-way ANOVA, *p*-value = 0.36). Some cross-batch differences were found in estimates of microbiome alpha diversity (one-way ANOVA, *p*-value_chao1_ = 0.02; *p*-value_shannon_ = 0.12; *p*-value_PD.whole.tree_ = 0.10; *p*-value_observed.species_ = 0.02) and beta-diversity (PERMANOVA, *p*-value_bray_curtis_ = 0.001; *p*-value_w.unifrac_ = 0.001; *p*-value_u.unifrac_ = 0.004). On the other hand, there were no associations between the batches and participants’ clinical characteristics including FIT status (permutation-based chi-square test, *p*-value = 0.76), CRA- vs CRA + (permutation-based chi-square test, *p*-value = 0.04), and high-risk CRA/CRC vs all others (permutation-based chi-square test, *p*-value = 0.74). Because the participants were generally balanced across batches, we did not adjust for batch in our primary analyses, which may have generated false positive discoveries as well as reducing statistical power.

**Figure 4:**
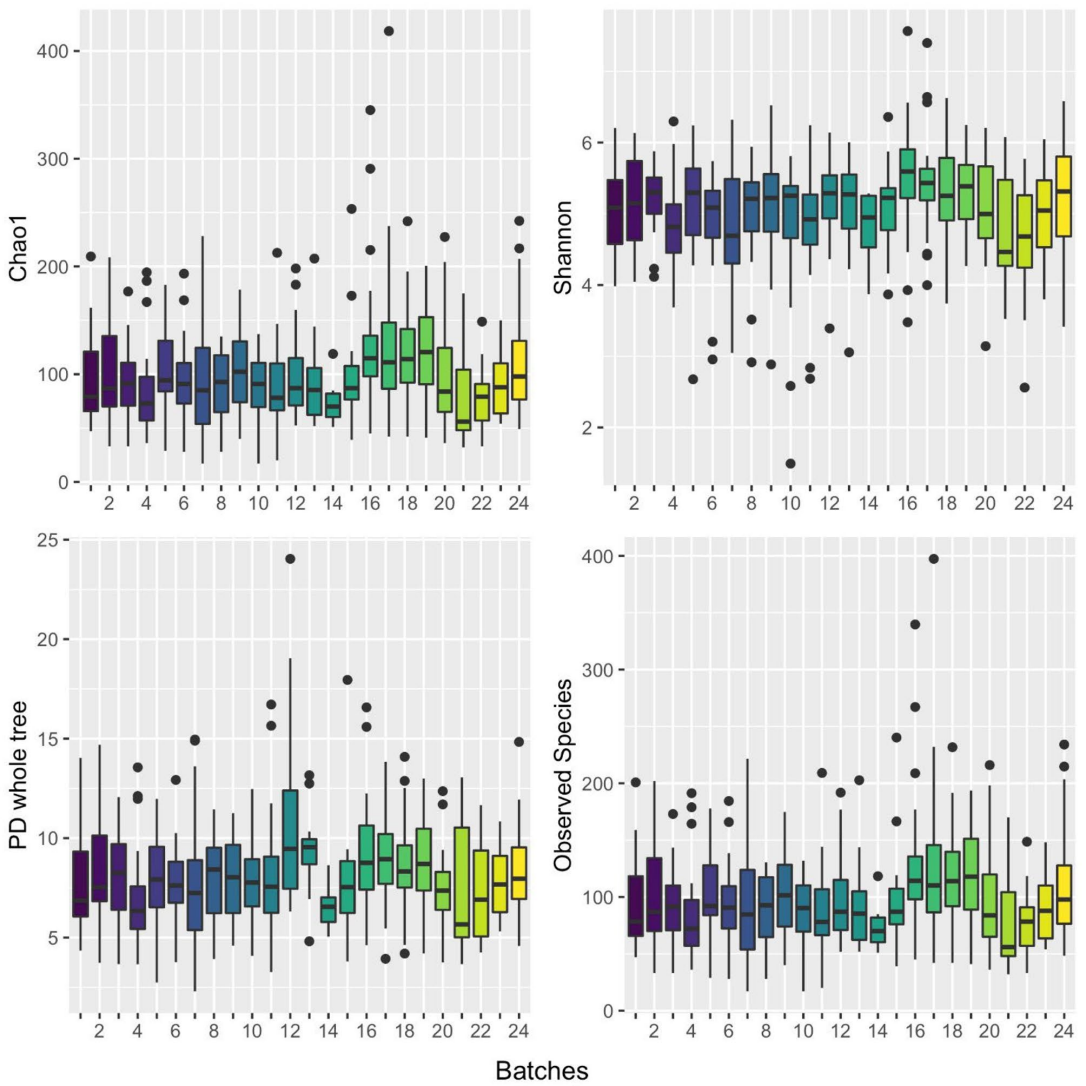
Estimated richness (Observed Species) and three estimates of alpha diversity (Chao1, Shannon, and PD.whole.tree) are presented across all 24 batches of the 72 quality control (QC) samples. For each box, the central line indicates the mean value, the box is the interquartile range (IQR, 25th to 75th percentile), the whiskers are the largest (or smallest) value within 1.5-times the IQR), and outlier values are dots. Box colors are arbitrary and have no particular meaning.

The 72 QC samples were of three types: Artificial Colony, Robogut A and Blank. As each batch contained at most one QC sample of each type, no statistical analysis was performed. Rather, all QC richness and alpha diversity estimates across the 24 batches are presented in Fig. 4. Principal coordinate plots of beta diversity estimates for the QC samples are shown in Figure S3. Batches 11 and 18 had elevated alpha diversity (and beta diversity outliers) in the Blank QCs, and batches 23 and 24 had elevated alpha diversity (and beta diversity outliers) in the Artificial Colony QCs, suggesting that these batches might be systematically different than others. Thus, we conducted the following sensitivity analyses.

### Sensitivity analyses

Microbiome alpha and beta diversity associations with FIT and CRA/CRC status were repeated after removing the four batches with outlier QC values. As in the primary analysis (Table 2), Chao1 and Shannon estimates of alpha diversity were higher in FIT+ than FIT−, whereas Shannon was higher in CRA + than CRA-neg participants (Table S2). In beta diversity analyses by MiRKAT, FIT status was associated with Bray–Curtis dissimilarity (*P* = 0.02) but not with W.UniFrac (*P* = 0.35) or U.UniFrac (*P* = 0.39); Omnibus test *P* = 0.059. Likewise CRA + status was associated with Bray–Curtis dissimilarity (P = 0.012) but not with W.UniFrac (*P* = 0.49) or U.UniFrac (*P* = 0.86); Omnibus Test *P* = 0.030). Thus, excluding the four outlier batches, these sensitivity analyses yielded results nearly identical to results of the primary analyses.

## Discussion

This is the first microbiome study of FIT devices after they have been used for at-home fecal sampling, postal shipping, and laboratory-based automated detection of heme for primary CRA/CRC screening in a large, fully integrated health plan. As per standard clinical and institutional practice, FIT+ (that is, heme+) participants were referred for colonoscopy to identify large-bowel pathology, particularly CRA and CRC. Per protocol, we enriched the study population by including all FIT+ and a small, random sample of FIT-negatives. With a mean age of 62, most participants in our comprehensive pre-paid health program had been screened for CRA/CRC previously. Thus, among the FIT+ we found the expected proportions with CRC (1.7%), high-risk CRA (14.9%), and low-risk CRA (22.5%). Also as expected, we found that men had an approximately twofold higher risk of CRA/CRC than women.

We found virtually no microbiome associations with CRA/CRC, in contrast to published studies based on feces collected specifically for microbiome and related molecular analyses^6–16,21^. The reasons for this discrepancy may be many. Foremost, we would postulate that the quantity of feces obtained by many participants, while adequate for heme detection, may have been inadequate to generate 16S rRNA-based amplicons representative of the fecal microbiota. Although we could not quantify total feces in each of the 980 FIT collected routinely by our members, we found that 61% of them yielded satisfactory microbiome profiles with stringent (10,000-read) rarefaction and 92% had satisfactory profiles with more lenient (1000-read) rarefaction. While likely useful for some research applications, these yields are lower than the excellent microbiome metrics obtained with FIT collected by clinical and research staff^19–21^.

Second, our specimens may have degraded, as our participants did not immediately freeze or chemically stabilize their FIT devices at home, although we note that upon receipt they were expeditiously tested for heme, then frozen immediately at −20 °C, and maintained thereafter at or below −70 °C until DNA extraction, amplification, and sequencing. Sinha and colleagues reported that microbiome metrics were highly stable in FIT stored at room temperature for four days^22^. Consistent with that, rarefaction at 10,000 reads in the current study yielded the expected distributions of alpha diversity (1016 species-level ASVs) and taxonomy (46 genera).

Third, true associations might have been attenuated by contamination as suggested by our blank-QC FIT devices. Consistent with Rounge and colleagues’ findings of possible cross-contamination by the FIT detection system^23^, the contamination in our specimens was at low level and in only two of 24 batches. Moreover, our positive control QCs performed as expected in nearly every batch (24 of 24 with Robogut A, and 22 of 24 with Artificial Colony). Sensitivity analyses still yielded no CRA associations when the affected batches were excluded.

Fourth, we had no statistical power to replicate previously reported microbiome associations with CRC^8–11^, and we may have had insufficient power to detect generally weaker associations with CRA^7,12–16^. And lastly, researchers and journals may have been reluctant to publish null fecal microbiome associations like ours.

Beyond our failure to identify FIT-based fecal microbiome associations with CRA, our study had several noteworthy strengths and a few additional weaknesses. KPH is an integrated health system, with (pre-COVID) 80–90% participation rate in annual FIT screening. Thus, our participants are highly representative of the underlying population with respect to age, sex, and race. KPH’s comprehensive EMR afforded assessment of demographics, diabetes, overweight/obesity, and use of antibiotics as potential confounders. And KPH provided colonoscopic and histopathologic ascertainment, at the expected rates, of CRC, high-risk CRA, and low-risk CRA, despite possible underascertainment due to incomplete colonoscopies. We had no participants at very high CRC risk, such as inflammatory bowel disease or hereditary polyposis, as such patients are monitored by colonoscopy, not with FIT screening. We classified our FIT-negatives as negative for CRA/CRC, which is justified by Kaiser Permanente Northern California’s report that a single FIT had 97.9% negative predictive value for CRA^24,25^. We also classified as CRA-negative participants who were found to have trivially small polyps or rare extraneous conditions. Clinical and laboratory staff were masked to each other’s data at all steps.

## Conclusions

We demonstrated that bar-coded FIT devices can be systematically frozen immediately after screening for fecal heme, and that fecal DNA in these used devices can be extracted, amplified, and sequenced to generate microbiome profiles for association analyses and other research applications. We found no clear associations with CRA/CRC with either stringent (10,000-read) or lenient (1000-read) rarefaction. These findings should be considered for the impact on statistical power and sample sizes of future prospective studies designed to understand the relationship of the fecal microbiota to CRA/CRC. For broad, large-scale success in reducing CRC-related morbidity and mortality, technical issues with fecal screening (including quantity, stabilization, shipping, and storage); the potential adverse impact on population-level participation rate; and the value and costs of cutting-edge laboratory methods such as metagenomics, meta-transcriptomics, and metabolomics must be resolved.

## Methods

### Field site and CRA/CRC screening

Kaiser Permanente Hawaii (KPH) is a fully integrated health plan that encourages all members age 50–75 (excluding those with previous CRA or CRC, who are followed by colonoscopy) to participate in annual CRA/CRC screening^2^; approximately 75% of these members provide a FIT for screening each year. Thus, members with previous CRA or CRC were excluded from the current study, but those with previous FIT screening (irrespective of FIT result) were eligible. Colonoscopy is not used for routine screening. The first stage of screening entails self-collection, at home, of feces using a licensed, commercially available, barcode labeled FIT device (Polymedco OC-Auto Micro FOB Test, Cortland Manor NY). The device generally obtains 10–50 mg of feces in a sealed vial containing 2 mL of proprietary solution. Within 3 days, it is sent at ambient temperature in a prepaid mailer through the US Postal Service to the KPH reference laboratory (Moanalua Medical Center) where, on the next business day, immunochemical testing for human hemoglobin is performed with a dedicated robotic instrument system (Polymedco Auto Sensor Diana) that is integrated with the laboratory’s information system. The positive cutoff with the Polymedco process and equipment is 100 ng hemoglobin / mL (stool or diluent), yielding an analytic sensitivity of 96.11%, specificity 99.33% (accessdata.fda.gov/cdrh_docs/reviews/K041408.pdf). Positive (FIT+) as well as negative results thus flow electronically from the instrument into the KPH electronic medical record (EMR). Patients with a FIT + result are referred to KPH gastroenterology for second-stage screening by colonoscopy, which is usually completed within a few weeks. Descriptive colonoscopy results are added to the EMR on the same day. Gross and histopathologic diagnoses of biopsies and excised lesions are added within a week. The KPH primary care provider coordinates follow-up and clinical management, if required. All methods and procedures in the study were performed in accordance with the relevant guidelines and regulations.

### Background data

During the six-month interval March through August 2014, KPH tested 18,061 FIT devices (18,001 patients), of which 941 (5.21%) were FIT + (in 937 patients). Of the FIT + patients, colonoscopy revealed that 10 had CRC (ICD-9 153) and 171 had CRA (ICD-9 211.3) including 111 with tubular adenoma, 9 with tubovillous adenoma, and 15 with both tubular and tubulovillous adenoma. During 2014, the prevalence of type 2 diabetes was 19% and the prevalence of obesity [body mass index (BMI) > 30 was 22% in KPH members over age 50.

### Outcome and covariate classification

The current project employed a hierarchy of histopathologic diagnoses: first, CRC (excluding in-situ) if present; else high-risk CRA (at least one adenoma with diameter ≥ 1 cm or with villous histology); else low-risk CRA (< 1 cm diameter and no villous histology); else all other (including miscellaneous and benign). BMI, calculated from weight and height (Kg/M^2^), was classified as healthy (18.5–24.99), overweight (25–29.99), or obese (≥ 30). Race and ethnicity (self-declared), Charlson morbidity index, and medical diagnoses were ascertained from KPH EMR data. Races were grouped into four categories: White, Asian, multiple, and other/ambiguous race. Charlson morbidity index was classified into three categories: = 0; ≥ 1 and ≤ 3; ≥ 4.

Using the KPH electronic pharmacy records, we assessed oral and parenteral medications prescribed to our study participants during the 365 days prior to FIT collection. The medications included antibiotics (specifically, cephalosporins, fluroquinolones, macrolides, penicillins, tetracyclines, and aminoglycosides), cardiovascular drugs (specifically, statins, fibrates, beta blockers, calcium channel blockers, and other antihypertensives), corticosteroids, and proton pump inhibitors. Antibiotics and cardiovascular drugs were categorized by cumulative length of prescription (none = 0, < median = 1, ≥ median = 2). Corticosteroids and proton pump inhibitors were categorized as none vs any.

### Collection and shipping of specimens

Over the course of six months, all FIT + devices, plus four randomly selected FIT-negative devices per week (N = 96 total FIT-negatives), plus 24 blank FIT devices (run through detection system with no feces) were immediately frozen at −20 °C, then shipped on dry ice by overnight courier in approximately equal sized batches to the National Cancer Institute (NCI) repository in accordance with International Airline Transport Association (IATA) regulations. KPH staff excluded no specimens, as they knew only the FIT result, not the indication for testing or other information. The NCI repository organized the specimens into 24 batches, each including a blank FIT, an Artificial Colony specimen, and a Robogut A specimen^26^.

### Processing of specimens; generation and editing of 16S rRNA sequence data

At the Institute for Genome Sciences, University of Maryland School of Medicine, all 2 mL proprietary solution plus feces was suctioned from each FIT device. This was mixed with 350 µL of lysis buffer composed of 0.05 M potassium phosphate buffer containing 50 µL lyzosyme (10 mg/mL), 6 µL of mutanolysin (25,000 U/ml; Sigma-Aldrich) and 3 µL of lysostaphin (4000 U/mL in 98sodium acetate; Sigma-Aldrich, St. Louis, MO). The mixture was incubated for 1 h at 37 °C, following which 10 µL proteinase K (20 mg/ml), 100 µL 10% SDS, and 20 µL RNase A (20 mg/ml) will be added. This mixture was incubated for 1 h at 55 °C. To further lyse microbial cells, Lysing Matrix B 2 ml beads [MP Biomedicals (Santa Ana, CA)] was added, following which mechanical disruption (bead beating) was performed on the mixture using a FastPrep instrument (MP Biomedicals, Solon, OH) set at 6.0 m/s for 30 s. The lysate was processed using the QIAsymphony SP protocol Pathogen complex 400 (Qiagen, Gaithesburg, MD) according to the manufacturer’s recommendation. The DNA was eluted into 100 µL of storage buffer [QIAsymphony reagent buffer AVE (0.04% sodium azide), Qiagen], pH 8.0. PCR inhibitors were removed from the extracted DNA using the Zymo-Spin IV Spin Filter column according to the manufacturer’s recommendations (Irvine, CA). DNA was quantified by Quant-iT PicoGreen (Molecular Probes, Inc., Eugene, OR) in a SpectraMax M5 microplate reader (Molecular Devices, Sunnyvale, CA).

A region of approximately 469 bp encompassing the V3 and V4 hypervariable regions of the 16S rRNA gene was targeted for sequencing. This region provides ample information for taxonomic classification of microbial communities from specimens associated with human microbiome studies and was used by the Human Microbiome Project^27^. Fusion dual barcoded primers 319F (5’ ACTCCTACGGGAGGCAGCAG-3’) and 806R (5’-GGACTACHVGGGTWTCTAAT- 3’) were used to amplify the V3–V4 region of bacterial 16S rRNA genes. The amplicons were pooled in equimolar concentration and sequenced on an Illumina MiSeq Instrument using the 300 bp paired-end protocol. The sequenced reads were processed using the following steps: (1) removal of primer sequence, (2) truncation of reads not having an average quality of 20 over a 30 bp sliding window based on the phred algorithm ^28,29^ implemented previously ^30,31^, (3) removal of trimmed reads having less than 75% of their original length, and (4) removal of the mate of reads that were discarded for having less than 75% original length. The Quantitative Insights Into Microbial Ecology (QIIME pipeline, version 1.6.0)^32^ was used for all further sequence processing steps, including quality trimming and demultiplexing. Quality trimming in QIIME was performed using the following criteria: (1) truncate sequence before 3 consecutive low quality bases and re-evaluate for length, (2) no ambiguous base calls, and (3) minimum sequence length of 150 bp after trimming, (4) remove sequences with less than 60% identity to a pre-built Greengenes database of 16S rRNA gene sequences (Oct, 2012 version)^33^. Further data processing included denoising by clustering similar sequences with less than 3% dissimilarity using USEARCH^34^ and de novo chimera detection and removal in UCHIME v5.1^35^. Paired reads were stitched together with “N” between each sequence and processed as one sequence in the analysis.

### Informatics methods

The sequence raw files were demultiplexed by running split_libray_fastq command (QIIME 1.9.1)^32^ to extract forward reads and reverse reads. Then DADA2 pipeline was used to generate an OTU table and the related phylogenetic tree^36^. After quality filtering sequences and processing using error correction models, amplicon sequence variants (ASVs) were identified. Chimera sequences were also removed. For all samples including QC samples, there were an average of 15,960 reads/sample, and 22,065 sequence features were identified. Those sequence features were then aligned with the SILVA v128 database to get taxonomy information^37^. The data have been posted to the Sequence Read Archive (SRA) with Bioproject ID PRJNA673212: http://www.ncbi.nlm.nih.gov/bioproject/673212.

### Statistical analyses

To calculate microbiome alpha and beta diversities, we first rarefied all samples to 10,000 read counts so that all samples were comparable. Samples with sequencing depth less than 10,000 were removed. Analysis result at other rarefying level (5000 and 1000) is quantitively similar. Microbiome alpha diversities (Number of Species, Chao1, Shannon index and Phylogenetic diversity) and beta diversities (Bray–Curtis dissimilarity, weighted UniFrac distance and Unifrac distance) were calculated using phyloseq package in R (3.6.2)^38^ Associations between microbiome alpha diversities and disease diagnosis, and between demographic variables and disease diagnosis, were assessed through linear regression models or fisher exact test for continuous or categorical outcomes respectively. MiRKAT^39^ was used to assess the association between microbiome beta diversities and disease diagnosis. All statistical analysis was conducted in R (3.6.2).

For individual taxa analyses, we focused on the genus-level. We excluded rare amplicon sequence variants (ASVs, present in < 10% of specimens) and excluded specimens that had fewer than 1000 read counts. Wilcoxon rank-sum tests were used to compare FIT + vs FIT-negative and CRA + vs CRA-negative on relative abundance for each genus. In addition, linear regression with each genus’ relative abundance as the dependent variable was used to compare these FIT and CRA groups adjusted for age, sex, and race. Significance was declared at false discovery rate (FDR) of 0.05.

### Ethics approval and consent to participate

Prior to implementation, the project was reviewed and approved by the KPH Institutional Review Board. Only authorized KPH staff had access to personally identifiable data. For statistical comparisons to the fecal microbiome profiles, KPH provided to NCI a limited dataset that included demographics (sex, age, race/ethnicity); length of membership in KPH; FIT result; date of FIT; colonoscopy diagnosis and date; types of gastrointestinal surgery and dates; most recent height and weight; presence / absence of type 2 diabetes; medication prescriptions within 365 days of FIT; history of Crohns disease or ulcerative colitis, and other common, major clinical diagnoses. Based on analysis of coded data and specimens previously collected for clinical care, the NIH Office of Human Research Subjects Protection issued the determination (#12694) that the proposed project was not human subjects research as defined by 45 CFR 46 and thus was exempt from NIH Institutional Review Board review.

## Supplementary Information

Supplementary Information.

## Data Availability

The data have been posted to the Sequence Read Archive (SRA) with Bioproject ID PRJNA673212: http://www.ncbi.nlm.nih.gov/bioproject/673212. Artificial Colony and Robogut A quality control specimens may be available from Dr. Sinha (sinhar@exchange.nih.gov). Specimens from the primary FIT devices are no longer available.

## Abbreviations

CRA: Colorectal adenoma
CRC: Colorectal cancer
FIT: Fecal immunochemical test
QC: Quality control
KPH: Kaiser Permanente Hawaii
EMR: Electronic medical record
CI: Confidence interval
BMI: Body mass index
NCI: National Cancer Institute
IATA: International Airline Transport Association
QIIME: Quantitative Insights into Microbial Ecology
ASV: Amplicon sequence variant
FDR: False discovery rate
ABX: Antibiotics
