# Supplementary material for: Reusing a prepaid health plan’s fecal immunochemical tests for microbiome associations with colorectal adenoma
Source: Sci Rep. 2022 Aug 31;12:14801. doi: 10.1038/s41598-022-18870-w (PMC9433441; doi:10.1038/s41598-022-18870-w)
Supplement: Supplementary file 1 — Supplementary Information. [file 41598_2022_18870_MOESM1_ESM.pdf]

Supplementary Figure Titles and Legends

Reusing a prepaid health plan's fecal immunochemical tests for microbiome associations with colorectal adenoma

James J. Goedert, Zhenyi Wu, Cyndee H. Yonehara, Timothy B. Frankland, Rashmi Sinha, Gieira S. Jones, Yunhu Wan, Jacques Ravel, Ni Zhao, Stacey A. Honda.

Figure S1. Alpha diversities across all 24 batches. Estimated richness (observed species) and three estimates of alpha diversity (Chao1, Shannon, and PD.whole.tree) for all non-QC specimens distributed across 24 specimen batches. One-way analysis of variance (ANOVA) revealed no significant differences across the 24 batches.

Figure S2. Principal coordinate plots for three beta diversity estimates (Bray Curtis, weighted UniFrac and unweighted UniFrac) for 24 specimen batches.

Figure S3. Principal coordinate plots for three beta diversity estimates (Bray Curtis, weighted UniFrac and unweighted UniFrac) with 24 artificial colony, 24 blank, and 24 Robogut A quality control (QC) specimens.

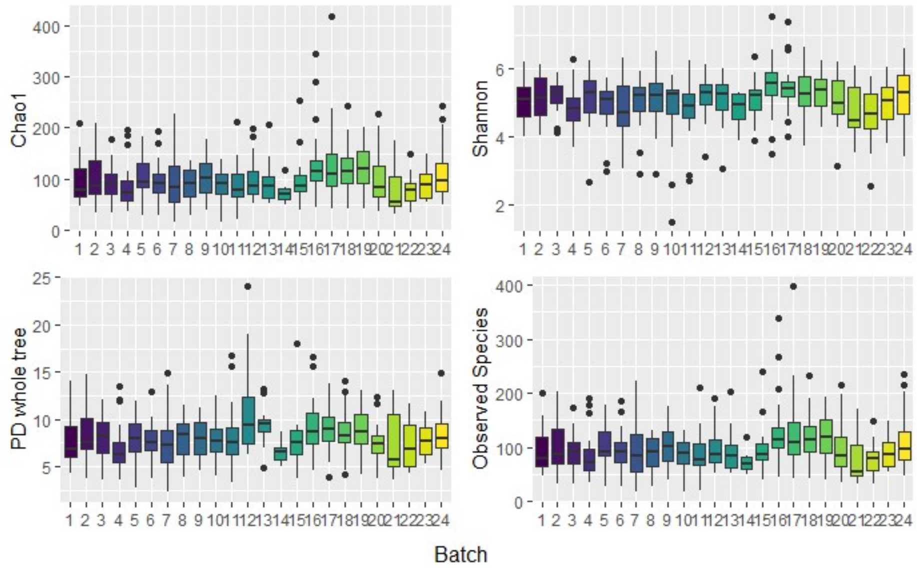

Figure S1. Alpha diversities across all 24 batches. Estimated richness (observed species) and three estimates of alpha diversity (Chao1, Shannon, and PD.whole.tree) for all non-QC specimens distributed across 24 specimen batches. One-way analysis of variance (ANOVA) revealed no significant differences across the 24 batches.

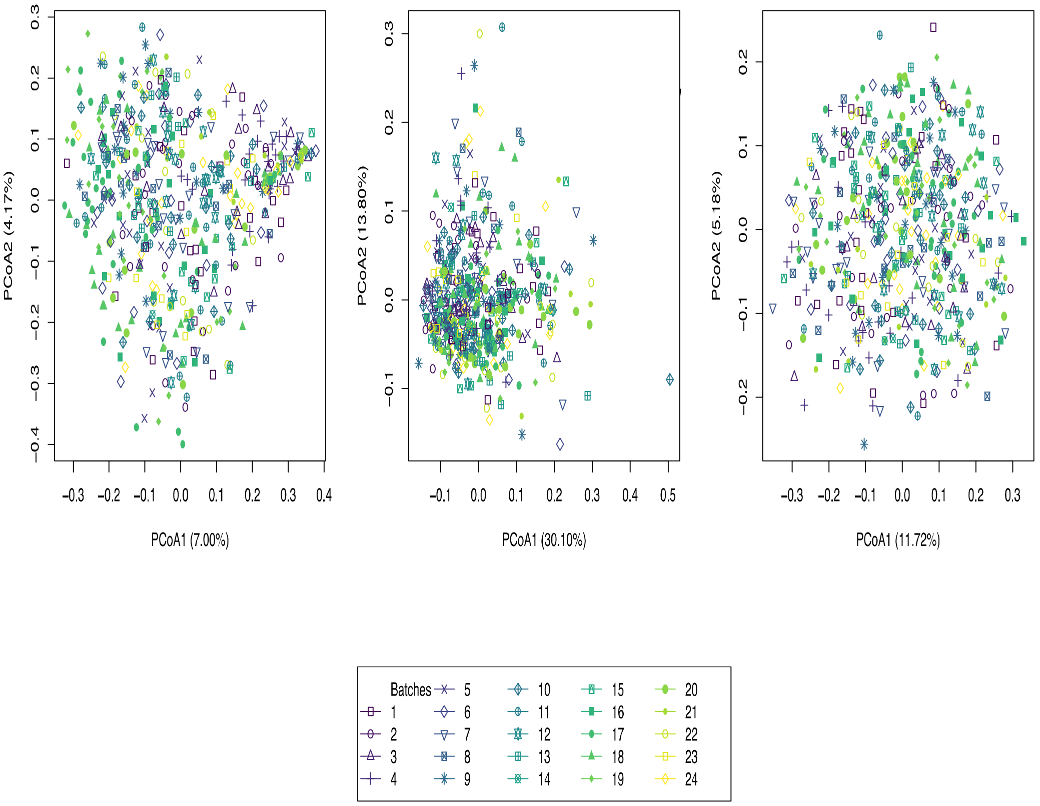

Figure S2. Principal coordinate plots for three beta diversity estimates (Bray Curtis, weighted UniFrac and unweighted UniFrac) for 24 specimen batches.

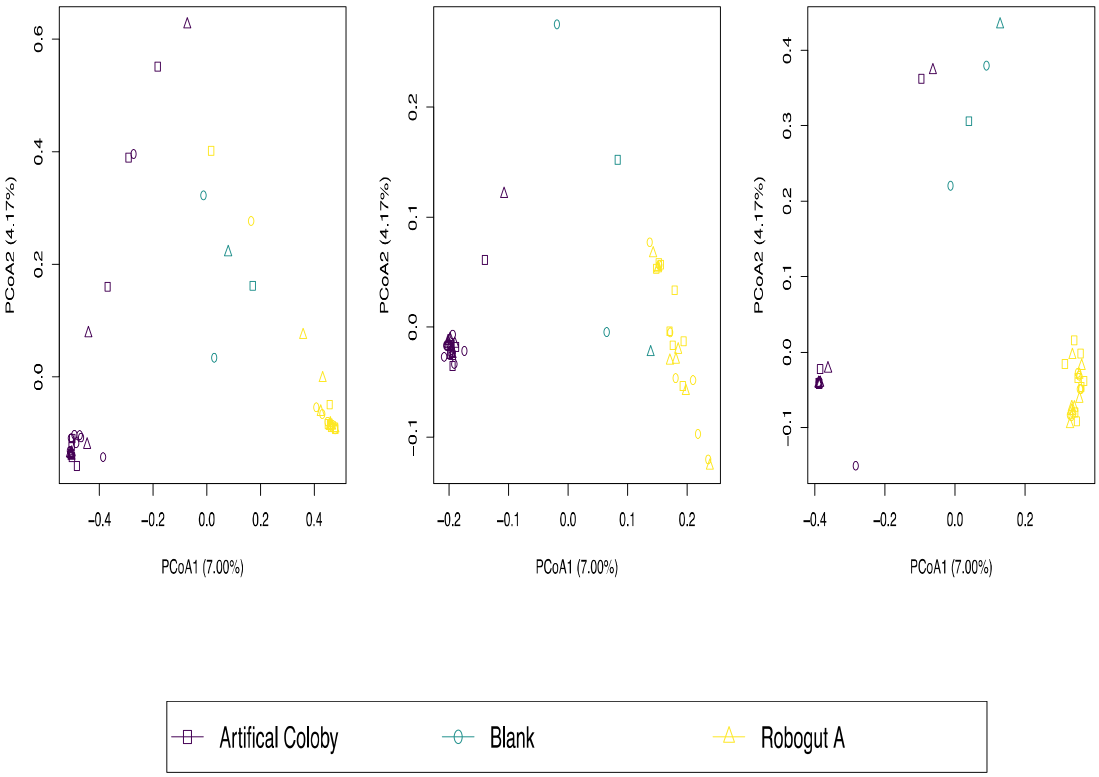

Figure S3. Principal coordinate plots for three beta diversity estimates (Bray Curtis, weighted UniFrac and unweighted UniFrac) with 24 artificial colony, 24 blank, and 24 Robogut A quality control (QC) specimens.

Supplementary Tables

Table S1. *Association between the logarithm of library sizes and clinical demographic variables. P-values were calculated from linear regression models with logarithm of library sizes as the response variable and the clinical/demographic variables as the independent variables.*

Variables	β (CI)	p-values
Age	0.004 (-0.008, 0.016)	0.523
Gender	-0.08 (-0.25, 0.08)	0.317
Race	-- [a]	0.118
High risk CRA/CRC	-0.020(-0.25,0.210)	0.868
CRA+	0.052 (-0.122, 0.226)	0.554
FIT +	-0.19(-0.44, 0.06)	0.142

[a] Because race had multiple categories, effect size (β) was not estimated.

Table S2. *Association between alpha diversities and diagnosis in sensitivity analysis (excluding batches 11, 18, 23 and 24)*

		FIT+ (390)				P-values[a]		
	FIT-	CRA-	Low risk CRA	High risk CRA	CRC	FIT- vs FIT+	CRA- vs CRA+	High risk CRA/CRC vs Other
#of Samples	50	242	86	55	7			
Alpha diversity: mean (SD)								
# of species	110.05(44.84)	94.64(42.84)	103.18(46.20)	100.78(42.63)	140.34(120.0)	0.09	0.10	0.30
Chao1	111.46 (46.42)	95.76(44.68)	104.73 (48.02)	101.83 (45.45)	144.61 (127.49)	0.10	0.10	0.30
Shannon	5.28(0.62)	5.00(0.74)	5.11(0.69)	5.18(0.60)	5.14(1.41)	0.04	0.06	0.25
PD	8.67(2.61)	7.92(2.57)	8.33(2.68)	8.37(3.13)	9.53(2.46)	0.16	0.14	0.29

[a] Linear regression.
